# LncRNA WFDC21P interacts with SEC63 to promote gastric cancer malignant behaviors by regulating calcium homeostasis signaling pathway

**DOI:** 10.1186/s12935-024-03297-2

**Published:** 2024-03-25

**Authors:** Jinyao Dong, Yongqiang Lv, Debin Meng, Ruyi Shi, Feng Li, Rui Guo, Yi Wang, Jiansheng Guo, Yanyan Zhang

**Affiliations:** 1 Hepatobiliary Pancreatogastric Surgery, Shanxi Cancer Hospital/Shanxi Hospital Affiliated to Cancer Hospital, Chinese Academy of Medical Sciences/Cancer Hospital Affiliated to Shanxi Medical University , Taiyuan, Shanxi 030013 P. R. China; 2 Scientific Research Department, Shanxi Cancer Hospital/Shanxi Hospital Affiliated to Cancer Hospital, Chinese Academy of Medical Sciences/Cancer Hospital Affiliated to Shanxi Medical University, Taiyuan, Shanxi 030013 P. R. China; 3 Department of Cell biology and Genetics, Shanxi Medical University, Taiyuan, Shanxi 030001 P. R. China; 4 Central Laboratory, Shanxi Cancer Hospital/Shanxi Hospital Affiliated to Cancer Hospital, Chinese Academy of Medical Sciences/Cancer Hospital Affiliated to Shanxi Medical University, Taiyuan, Shanxi 030013 P. R. China; 5 Gastrointestinal Surgery Department, First Hospital of Shanxi Medical University, Taiyuan, Shanxi 030001 P. R. China; 6 Hepatobiliary Pancreatic Surgery and Liver Transplantation Center, First Hospital of Shanxi Medical University, Taiyuan, Shanxi 030001 P. R. China

**Keywords:** LncRNAs, WFDC21P, SEC63, Gastric cancer, Calcium homeostasis

## Abstract

**Background:**

Gastric cancer is currently estimated to be the fifth leading common cancer in the world, and responsible for about one million new cases and an estimated 769,000 cancer-related deaths each year. WFDC21P is long non-coding RNA and has been reported to play critical roles in serval types of cancer. Our research aims to investigate the biological effects and molecular mechanism of WFDC21P in gastric cancer.

**Methods:**

Datasets (GSE53137, GSE58828, and GSE109476) in GEO database were used to screen differential expressed lncRNAs in gastric cancer by online GEO2R analysis tool. Quantitative RT-PCR was used to verify the above prediction in ten pairs of gastric cancer and corresponding paracancerous tissues. Pan-cancer analysis was used to analyze the expression of WFDC21P in different types of cancer. Small interfering RNAs were used to WFDC21P knockdown. CCK-8 and colony formation assays were used to measure the proliferation and tumorigenesis abilities. Wound healing and Transwell assay were used to detect the migration and invasion abilities. Proteins that interact with WFDC21P were predicted by catRAPID database. RNA pull down and RNA Immunoprecipitation were used to confirm the interaction. Western blotting was used to detect the key proteins level in calcium homeostasis signaling pathway. Loss-of-function and rescue assays were used to evaluate the biological function of SEC63 at the background of WFDC21P silencing.

**Results:**

WFDC21P was upregulated in gastric cancer tissues and cell lines. WFDC21P downregulation suppressed proliferation, tumorigenesis, migration, invasion, and promoted apoptosis in gastric cancer. SEC63 protein had the capability to bind with WFDC21P and the expression of SEC63 was regulated by WFDC21P. SEC63 was also upregulated in gastric cancer and exerted effects during tumor growth and metastasis.

**Conclusions:**

This study confirmed that lncRNA WFDC21P aggravated gastric cancer malignant behaviors by interacting with SEC63 to regulate the calcium homeostasis signaling pathway.

**Supplementary Information:**

The online version contains supplementary material available at 10.1186/s12935-024-03297-2.

## Background

Gastric cancer (GC) is the fifth leading prevalent malignant tumor and fourth for mortality, which is responsible for more than one million new cases and an estimated 769,000 cancer-related deaths globally [1]. Nowadays, stomach cancer is the third most prevalent tumor and cancer-related deaths in China, because H. pylori infection is still high, infecting more than half of the people. Other risk factors like alcohol consumption, smoking, low fruit intake, and the consumption of preserved food by salting also contribute to high prevalence of gastric cancer [2, 3]. Although the development of systemic therapies, including standard radical gastrectomy, immunotherapy, and chemotherapy, patient with gastric cancer still have poor prognoses because of lacking biomarkers for early diagnosis, high frequency of lymphatic metastasis, and recurrence after surgery [4]. Therefore, investigating the molecular pattern and regulatory networks underlying the tumorigenesis and progression of GC is critical.

Long non-coding RNAs (lncRNAs) are a subset of non-coding RNAs (ncRNAs), which were initially considered mere byproducts of gene transcription [5]. With the development of gene microarray, high-throughput sequencing, and bioinformatic tools, an increasing number of lncRNAs are being noticed [6]. Accumulating evidence has demonstrated that lncRNAs were involved in regulating the occurrence and development of diseases, particularly in tumorigenesis and progression [7]. For example, lncRNA AK002107 acted as a competing endogenous (ceRNA) to sponge miR-140-5p, inducing epithelial–mesenchymal transition in hepatocellular carcinoma by targeting TGFBR1 [8]. In gastric cancer, CAFs-derived exosomal DACT3-AS1 is a suppressor in malignant transformation and ferroptosis-mediated oxaliplatin resistance [9]. A hypoxia-induced lncRNA-CBSLR interacts with m6A “reader” YTHDF2 to protect GC cells from ferroptosis, leading to chem-resistance by forming a CBSLR/YTHDF2/CBS signaling axis that decrease the stability of CBS mRNA [10]. Above evidences suggests that lncRNAs is not simply a noise of gene transcription but also take important effects in regulating tumor malignant behaviors and cancer therapies. Nevertheless, only a small number of lncRNAs in GC have been investigated.

The SEC protein family, including SEC61, SEC62, and SEC63, has been reported to play a pivotal role in protein translocation [11]. Apart from protein translocation functions, SEC61 was widely reported to regulate the intracellular Ca^2+^ homeostasis [12, 13]. Recent years, study has indicated that SEC62 was a regulator of ER stress in eukaryotic cells, which plays an important role in human endoplasmic reticulum (ER) microenvironment [14]. Although the SEC61 and SEC62 have been investigated, the role of SEC63 in human biological and pathological processes remain elusive. There were many studies indicated that SEC63 play a crucial role in regulating serval types of gastrointestinal tumors, including bowel cancers [15], colorectal cancers [16], and hepatocellular carcinoma [17]. In this study, we screened lncRNAs microarray datasets in the Gene Expression Omnibus (GEO) database. After bioinformatic analysis and qPCR verification, lncRNA WAP four-disulfide core domain 21 pseudogene (WFDC21P) was found to be upregulated in GC tissues than in the corresponding adjacent nontumorous tissues. Loss-of-function studies identified that WFDC21P promotes tumor growth and metastasis in GC. Further mechanism studies reveal that WFDC21P interacted with SEC63 protein to promote tumor malignant behaviors by regulating calcium signaling pathway. Our findings suggest that lncRNA WFDC21P and SEC63 may serve as new biomarkers and therapeutic targets in patients with GC.

## Materials and methods

### Cell lines and tissues

Human gastric cancer cell lines (AGS, N87) were purchased from the American Type Culture Collection (ATCC), MNK-45 and SGC-7901 were obtained from the German Collection of Microorganisms and Cell Cultures GmbH (DSMZ). Human normal gastric cell line (GES-1) and human 293T cells were purchased from ATCC. Cell lines were cultured in Dulbecco’s modified Eagle medium (DMEM; Sigma-Aldrich, USA), plus 10% fetal bovine serum (FBS; Gibco, USA). Cells were maintained at 37 ℃ with 5% CO_2_. Human gastric cancer and corresponding paracancerous tissues were obtained from Hepatobiliary Pancreatogastric Surgery, Shanxi Cancer Hospital in February 2022. Tissues were immediately soaked into RNA later (beyotime, China) after excision. All tissues were saved at -80 ℃ until used. Following criteria were excluded: (1) patients younger than 18 years, older than 75 years, and without full civil capacity; (2) patients who were diagnosed as cancer in other organs; (3) patients received anti-cancer therapies before surgery. This study was approved by the ethics committees of Cancer Center of Shanxi province. Informed consents were obtained from all participants.

### Bioinformatic analysis

LncRNAs microarray data about GC (GSE53137, GSE58828, and GSE109476) were downloaded from Gene Expression Omnibus (GEO) database. Online GEO2R tool was used to screen the differential expression lncRNA between GC and normal gastric tissues in each dataset, the thresholds were set as *p* < 0.05 and fold change > 2 or fold change < 2. After that, we used online Veen tools [18] to take the intersection of above three datasets for narrowing the range of upregulated and downregulated lncRNAs in GC and adjacent normal tissues. catRAPID database [19] were used to screen the proteins that can interact with indicated lncRNAs.

### Total RNA extraction, reversed transcription, and quantitative real-time PCR

Total RNAs from tissues and cells were extracted using TRIzol (Invitrogen, USA) regent according to standard protocol. PrimeScript™ RT reagent Kit (Takara, China) was used to synthesis complementary DNA (cDNA), TB Green® Premix Ex Taq™ II (Takara, China) was used to performed quantitative real-time PCR (qPCR). The expression level of RNAs were normalized by Glyceraldehyde-3-phosphate dehydrogenase (GAPDH). The 2^−ΔΔCt^ method was used to determine the expression of indicated RNAs. Primer sequences were listed in Supplementary Table S1.

### Western blotting

Western blotting was performed according standard protocol. Briefly, total proteins from the cells or tissues were extracted using the Sodium dodecyl sulfate (SDS) lysis buffer (Solarbio, China) plus proteinase inhibitor mix (Solarbio, China). According to their molecular masses, the proteins were separated by 10% or 12.5% polyacrylamide gels. After that, the proteins were transferred onto polyvinylidene difluoride membranes (Millipore, Germany). 5% skim milk was used to block the nonspecific binding for 1 h. The membranes were then incubated at 4 ℃ with indicated antibodies overnight. About 12–14 h next day, the membranes were washed using TBS plus 0.01% Tween-20 (TBST), and incubated with secondary antibodies according to their species at room temperature for 1 h. An ECL chemiluminescence kit (Sigma-Aldrich, USA) was used to detect the proteins signal. The antibodies used in this study were listed in Supplementary Table S2.

### Small interference RNA synthesis, plasmid construction and transfection

Small interference RNAs (siRNAs) targeting WFDC21P were synthesized by Tsingke Biotechnology (Guangzhou, China). SEC63 overexpression and corresponding empty vector were purchased from Hanheng Biotechnology (Shanghai, China). Lipofectamine 3000 (Invitrogen, USA) was used to transfection according to manufacturer’s instructions. Forty-eight hours later, transfection efficacy of lncRNAs and mRNAs were verified using qPCR. The protein level of SEC63 were determined by western blotting after 72 h. The siRNA sequences used in this study were listed in Supplementary Table S1.

### Cell proliferation and colony formation assays

Cell count kit 8 (CCK-8) assay was used to measure the proliferation of gastric cancer cell lines. Briefly, AGS and SGC-7901 at a density of 3 × 10^3^ cells/well were seeded into 96-well plates for five-day test, 6-wells replicated each day. Next day, CCK-8 regent (MedChemExpress, China) was added to cell cultures and incubated for 3 hours. Cell proliferation ability at the indicated time was measured by Multiskan™ FC (Thermo Scientific, USA) at absorbance of 450 nm. Gastric cancer cell lines (AGS and SGC-7901) at a density of 5 × 10^2^ cells/well were inoculated in 6-wells plates. Cell medium was changed every three days. Fortnight after inoculation, cell colons was fixed using methanol and stained with 0.1% crystal violet solution. Numbers of colons were counted. CCK-8 and colony formation assays were performed at least three independent experiments.

### Wound healing and transwell assays

Wound healing assay were used to determine the migration of gastric cancer cell lines. 1 × 10^6^ cells were seeded into 6-wells plates. Overnight, 1 mL pipette tip was used to create a scratch in the middle of each well. Cells migrate across the scratch was observed at 0 and 48 h after scraping. Five random fields were selected for statistical analysis. Transwell assays were used to measure the invasion and migration ability of gastric cancer cell lines. For invasion assay, Matrigel (Corning, USA) was used to pre-coat the membrane for 30 min at 37 ℃, target cells (5 × 10^4^ cells/chamber) were resuspended at basal medium and seeded in upper chamber. In migration assays, 4 × 10^4^ cells/chamber were seeded in upper chamber and maintained in basal Medium. DMEM plus 10% FBS was added in lower chambers for both invasion and migration assays. Thirty-six hours after inoculation, a cotton swab was used to remove the cells remaining in the upper polycarbonate layer. The chambers were subjected to methanol to fix the cells in lower polycarbonate layer for 10 min. After that, 0.1% crystal violet solution was used to stain the cells. The number of crystal violet-stained cells was counted in three random fields at 100× magnification. The wound healing and Transwell assays were performed at least three independent replications.

### RNA immunoprecipitation assay

RNA immunoprecipitation (RIP) was performed using an Imprint RNA Immunoprecipitation Kit (Sigma-Aldrich) according to the manufacturer’s instructions. Briefly, protein A magnetic beads was pre-mixed with anti-SEC63 antibody or anti-IgG for 2 h at room temperature. Cell lysis plus protease inhibitor cocktail and ribonuclease inhibitor were incubated with the mix (protein A magnetic beads and anti-SEC63 or IgG) at 4 ℃ overnight to perform immunoprecipitation. Next day, total RNAs were extracted by TRIzol regent, the enrichment of WFDC21P by SEC63 protein was measured using qPCR as described previously.

### RNA pull down

Pierce RNA 3´ End Desthiobiotinylation Kit (Thermo Scientific, USA) was used to synthesize biotinylated WFDC21P. Pierce™ Magnetic RNA-Protein Pull-Down Kit (Sigma-Aldrich, USA) was used to perform RNA pull down assay according to standard protocol. Labeled WFDC21P was enriched by streptavidin magnetic beads for 1 h at 37 ℃ and AGS and SGC-7901 cell lysis were added. The mix was incubated for 2 h at room temperature. Total proteins were extracted and the expression of SEC63 was detect by western blotting as described previously.

### Statistical analysis

GraphPad Prism 8.0 (La Jolla, USA) or SPSS 21.0 (IBM Corp, USA) were used to perform statistical analysis. Differences between groups were analyzed using Student’s t-test or Analysis of Variance. *p *< 0.05 was defined as statistical significance.

## Results

### WFDC21P was upregulated in gastric cancer tissues and cell lines

**Fig. 1:**
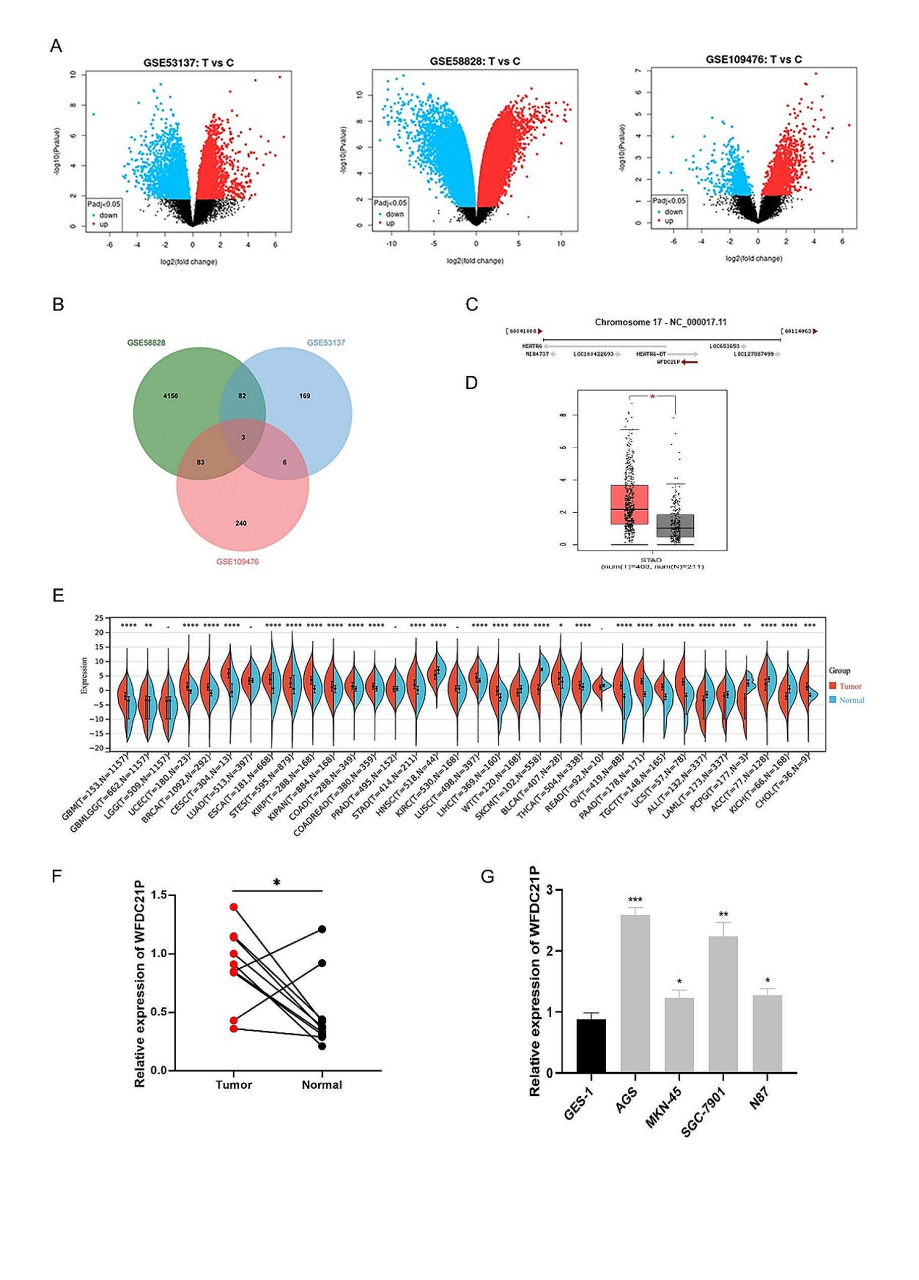
WFDC21P was upregulated in gastric cancer. (**A**) Volcano plot showed the differential expressed lncRNAs in three datasets (GSE53137, GSE58828, and GSE109476). (**B)** Veen diagram showed the common upregulation lncRNAs in above three datasets. **(C)** The structure of lncRNA WFDC21P in GEO database. **(D)** The expression of WFDC21P in TCGA database. **(E)** Pan-cancer analysis was performed to measure the expression of WFDC21P in different types of cancer. **(F)** qPCR assay was used to detect the expression of WFDC21P in 10 pairs of gastric cancer and corresponding paracancerous tissues. **(G)** qPCR assay was used to measure the expression of WFDC21P in gastric cancer cell lines (AGS, MKN-45, SGC-7901, and N87) compared to normal gastric cell lines (GES-1). The data are presented as the mean ± SD of at least three independent experiments. ns, not significant; *, *p* < 0.05; **, *p* < 0.01; ***, *p* < 0.001; ****, *p* < 0.0001

To investigate lncRNAs that affect the tumor growth and metastasis in gastric cancer, the online GEO2R tool was used to screen the gastric cancer microarray data in GEO database. Three datasets (GSE53137, GSE58828, and GSE109476) attracted our attentions (Fig. 1A). After bioinformatic analysis, three upregulated lncRNAs were identified in gastric cancer tissues than of adjacent normal tissues (Fig. 1B). Among the upregulated lncRNAs, a lncRNA which derived from WAP four-disulfide core domain 21 pseudogene (WFDC21P) locus was the most significant in GC tissues (Fig. 1C). Right after, GEPIA database was used to verify the expression of WFDC21P in gastric cancer. The results indicated that WFDC21P was significantly upregulated in GC (Fig. 1D). Take a step further, we performed Pan-cancer analysis using GEPIA database. The Pan-cancer analysis results showed that the expression of WFDC21P was various among different tumors (Figure. 1E). Therefore, qPCR was used to detect WFDC21P expression in 10 pairs of GC tissues and corresponding paracancerous tissues. The qPCR result showed that WFDC21P was upregulated in GC tissues than that in adjacent normal tissues (Fig. 1F). To further confirm this result, we screen the expression of WFDC21P in GC cell lines (AGS, MKN-45, SGC-7901, and N87) and normal gastric cell lines (GES-1). qPCR result indicated that WFDC21P expression was also upregulated in GC cell lines compare to normal gastric cell lines (Fig. 1G). Among GC cell lines, AGS and SGC-7901 were the most significant upregulated. Therefore, AGS and SGC-7901 were selected for further studies. Above results indicate that lncRNA WFDC21P was upregulated in both GC tissues and cell lines.

### WFDC21P promoted gastric cancer growth and metastasis

**Fig. 2:**
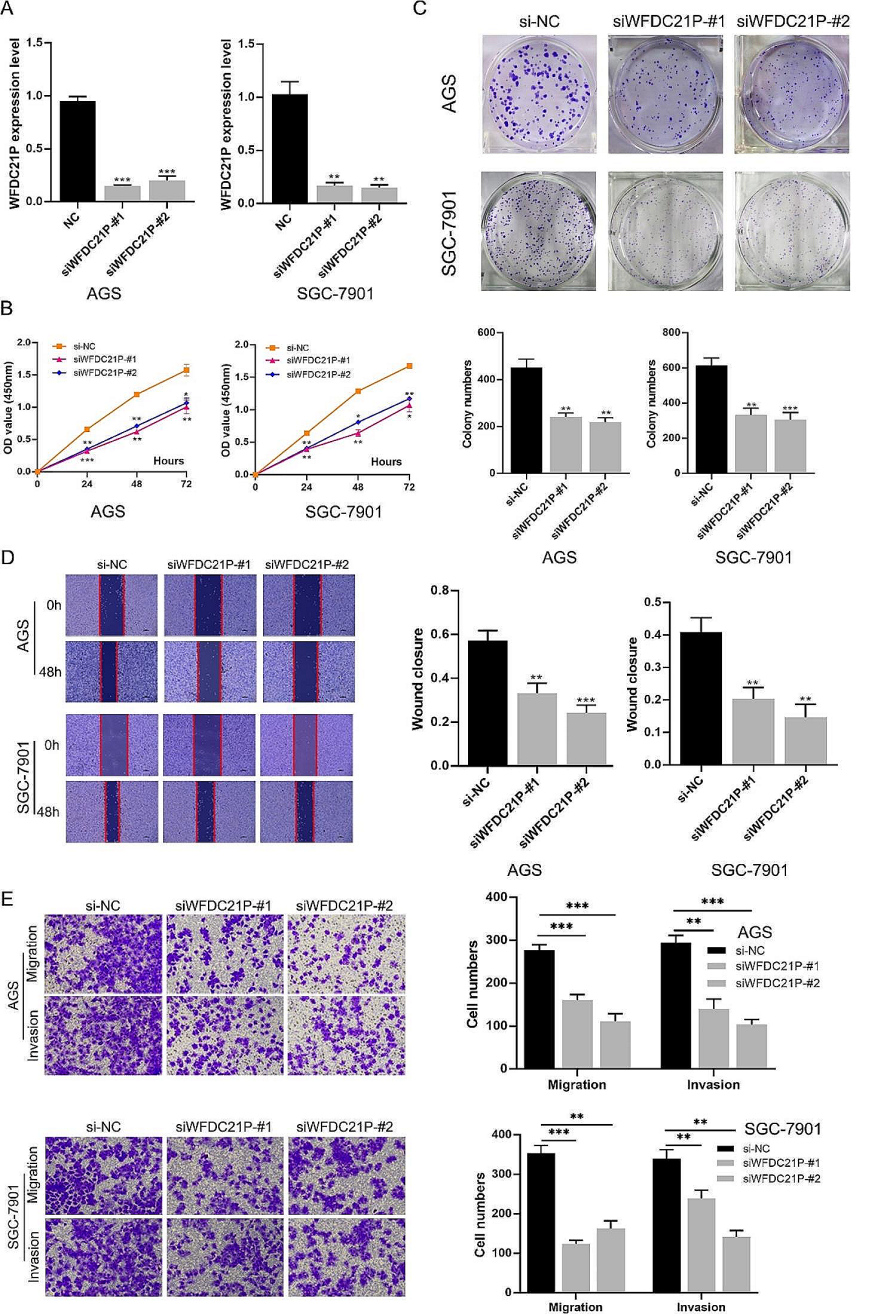
WFDC21P downregulation inhibited gastric cancer growth and metastasis. (**A**) Inhibition efficiency of siRNAs targeted WFDC21P were verified by qPCR assay. (**B**) CCK-8 were used to measure the tumor proliferation ability after WFDC21P downregulation. (**C**) Tumorigenesis ability was detected by colony formation assays after inhibiting WFDC21P expression. (**D-E.**) The change of migration and invasion abilities induced by WFDC21P downregulation were measured using wound healing and Transwell assays. The data are presented as the mean ± SD of at least three independent experiments. ns, not significant; *, *p* < 0.05; **, *p* < 0.01; ***, *p* < 0.001

An increasing number of studies have showed that lncRNAs may affect the tumor malignant behaviors [20–22]. In order to investigate the tumor growth and metastasis abilities affect by WFDC21P, small interference RNAs (siRNAs) target the WFDC21P sequence were used to inhibit the expression (siWFDC21P-#1 and siWFDC21P-#2). qPCR was used to verify the inhibition efficiency (siWFDC21P-#1 and siWFDC21P-#2) compare to negative control (si-NC) (Fig. 2A). CCK-8 assays were used to measure the proliferation abilities after inhibiting WFDC21P expression. The results indicated that WFDC21P downregulation significantly suppressed gastric cancer proliferation (Fig. 2B). Colony formation assays showed that inhibited the WFDC21P expression can suppress tumorigenesis (Fig. 2C). Furthermore, wound healing and Transwell assays were used to measure the migration and invasion abilities after inhibiting WFDC21P expression. The result of wound healing assays indicated that WFDC21P downregulation can inhibit gastric cancer migration (Fig. 2D). Transwell assays showed that inhibited the WFDC21P suppressed both migration and invasion (Fig. 2E). In summary, WFDC21P downregulation suppresses tumor growth and metastasis.

### WFDC21P interacted with SEC63 to regulate calcium homeostasis signaling pathway

**Fig. 3:**
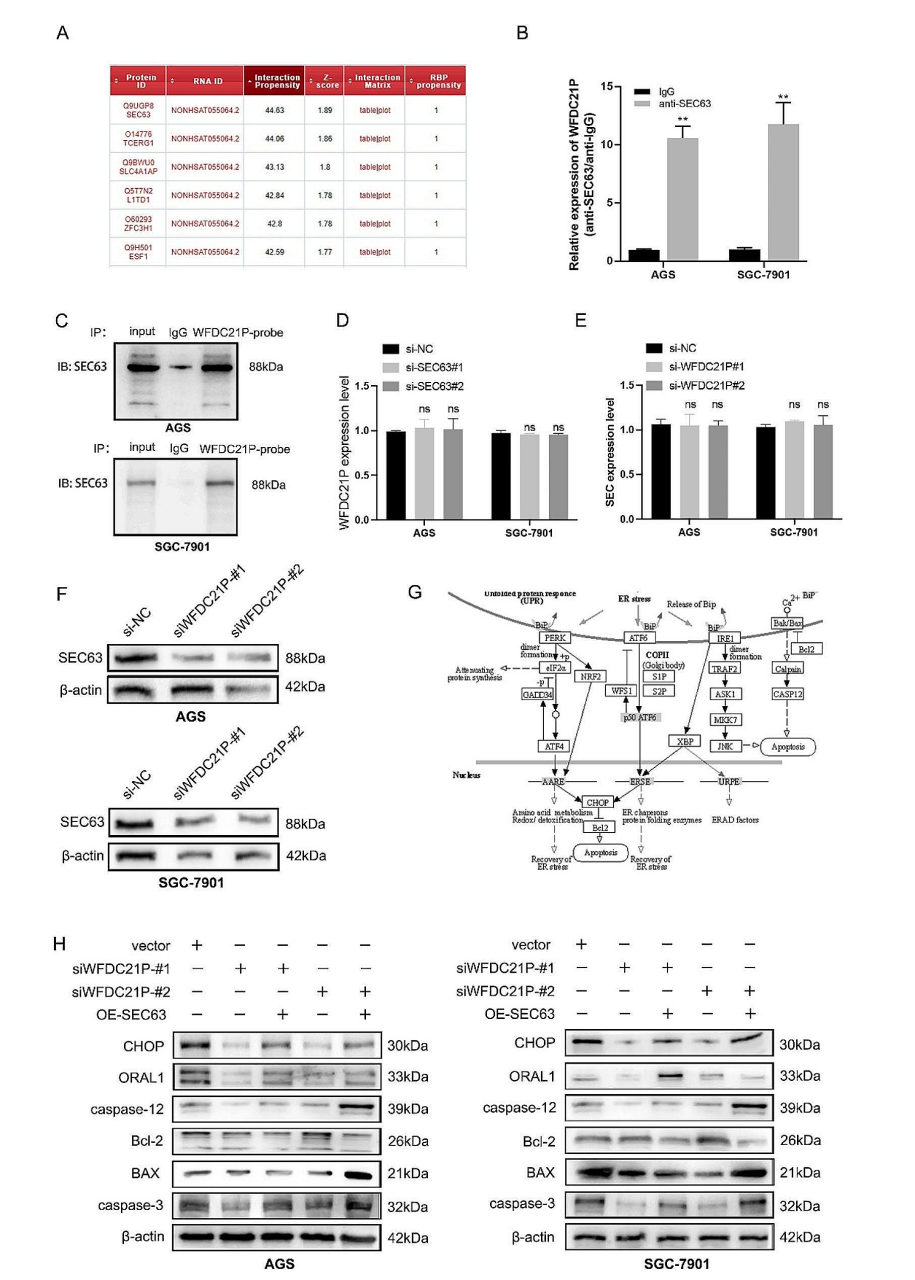
WFDC21P interacted with SEC63 to regulate calcium homeostasis signaling pathway. (**A**) Top five proteins that interacted with WFDC21P were predicted by catRAPID database. (**B**) RIP assays using anti-SEC63 antibody were performed to identify the interaction between WFDC21P and SEC63 protein. (**C**) Biotin labed WFDC21P sequence was used to performed RNA pull down. Western blotting was used to verify the enrichment of SEC63. (**D**) WFDC21P expression after inhibiting SEC63. (**E**) The mRNA level of SEC63 after inhibiting WFDC21P expression. (**F**) Western blotting assay was used to measure the SEC63 expression after WFDC21P downregulation. (**G**) KEGG chart showed the calcium homeostasis signaling pathway regulated by SEC63. (**H**) The key protein levels in calcium homeostasis signaling pathway were detected by western blotting after WFDC21P downregulation and SEC63 overexpression. The data are presented as the mean ± SD of at least three independent experiments. ns, not significant; **, *p* < 0.01

Previous studies have suggested that lncRNAs interacted with proteins to regulate tumorigenesis and progression [23–25]. Accordingly, we supposed that WFDC21P may also bind to proteins to regulate gastric cancer malignant behaviors. Thus, online bioinformatic tool catRAPID was used to predict the potential proteins that bind to WFDC21P. After bioinformatic analysis, SEC63 homolog, protein translocation regulator (SEC63) was predicted to have the capability to interact with WFDC21P (Fig. 3A). To further verified this prediction, RNA immunoprecipitation (RIP) and biotin labeled WFDC21P pull down were performed to detect the binding between WFDC21P and SEC63. The RIP result showed that WFDC21P were 10-fold more enrichment in anti-SEC63 rather than anti-IgG relative to 10% input (Fig. 3B). The RNA pull down assay using label WFDC21P also confirmed that SEC63 can bind to WFDC21P (Fig. 3C). Take a single step forward, we want to identify that SEC63 is upstream or downstream regulator of WFDC21P. Therefore, qPCR was used to detect the expression of WFDC21P after inhibiting the SEC63 expression using small interference RNAs targeted SEC63 sequence. The results indicated that SEC63 downregulation had no effect on the WFDC21P expression (Fig. 3D). Next, the expression of SEC63 was detected after WFDC21P downregulation, the results showed that the protein level of SEC63 was downregulated while the mRNA level remain unchanged (Fig. 3E-F). Above results determined that SEC63 was the downstream regulator of WFDC21P, and WFDC21P regulated the SEC63 expression at protein level. A substantial number of studies have reported that SEC63 was a critical protein which involved in calcium homeostasis regulation [12, 13, 26]. Therefore, we supposed that WFDC21P may regulate the calcium homeostasis signaling pathway by SEC63 mediated mechanism (Fig. 3G). Proteins that involved in calcium homeostasis regulation were measured using western blotting, the results indicated that WFDC21P downregulation inhibited the expression level of critical proteins in calcium homeostasis signaling pathway, however, this effect can be rescued by SEC63 overexpression (Fig. 3H). Above results show that WFDC21P regulates the cellular calcium homeostasis by interacting with SEC63.

**Fig. 4:**
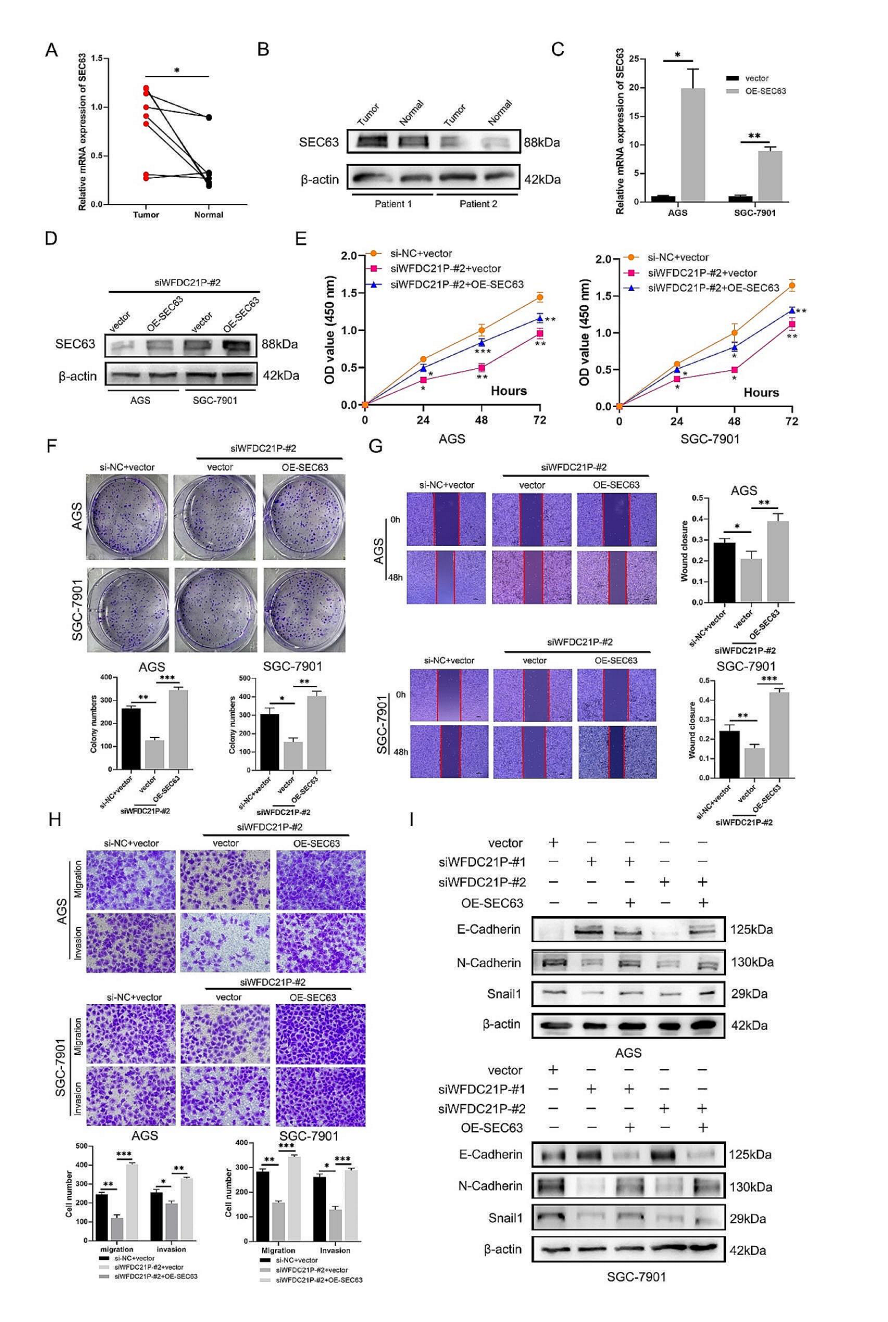
SEC63 promoted tumor growth and metastasis in gastric cancer. (**A**) The mRNA level of SEC63 in gastric cancer and corresponding paracancerous tissues was measured by qPCR assay. (**B**) Western blotting assay was used to detect the SEC63 protein level in gastric cancer and corresponding paracancerous tissues. (**C-D**). SEC63 overexpression plasmid transfection efficiency was verified by qPCR assay and western blotting assay respectively. (**E**). CCK-8 assay was used to measure the proliferation ability change induced by SEC63 overexpression. (**F**). Colony formation assay was used to measure the tumorigenesis ability affected by SEC63 overexpression. (**G-H**). Migration and invasion change induced by SEC63 overexpression were detectd by wound healing and Transwell assay at the context of WFDC21P inhibition. (**I**) The key protein levels in EMT were measured by western blotting after WFDC21P downregulation and SEC63 overexpression. The data are presented as the mean ± SD of at least three independent experiments. ns, not significant; *, *p* < 0.05; **, *p* < 0.01; ***, *p* < 0.001

### SEC63 played a critical role in promoting tumor malignant behaviors

To investigate the biological role of SEC63 in gastric cancer, GEPIA database was used to explore the SEC63 expression in tumor and normal tissues. The results showed that SEC63 was upregulated in gastric cancer compared to normal stomach tissues (Figure. S1). qPCR and western blotting showed that SEC63 was upregulated in gastric cancer tissues than that in adjacent normal tissues both at mRNA and protein level (Fig. 4A-B). To explore the SEC63 affected gastric cancer progression at the context of WFDC21P downregulation. qPCR and western blotting were used to verify the transfection efficiency of SEC63 overexpression plasmid (Fig. 4 C-D). CCK-8 results showed that SEC63 overexpression alleviated the anti-proliferation effect caused by WFDC21P inhibition (Fig. 4E). The results of colony formation indicated that SEC63 overexpression can increase the tumorigenesis in gastric cancer (Fig. 4F). While WFDC21P inhibition can suppress the migration and invasion abilities in gastric cancer, the wound healing and Transwell results showed that SEC63 overexpression rescued the anti-metastasis effect led by WFDC21P downregulation (Fig. 4G-H). Epithelial-Mesenchymal Transition (EMT) played an important role in tumor migration and invasion. Thus, western blotting was used to detect the key proteins in EMT. The results showed that WFDC21P downregulation inhibited N-cadherin and Snail1 but increase E-cadherin protein levels, however, this effect was rescued by SEC63 overexpression (Fig. 4I). Above results indicate that SEC63 overexpression suppresses the anti-tumor effect caused by interfering WFDC21P expression.

## Discussion

Despite advancements in systemic therapies for tumors [27], gastric cancer continues to pose a significant clinical challenge, characterized by a malignant nature and an overall poor prognosis [28]. Numerous studies have consistently demonstrated the profound influence of genetic and epigenetic backgrounds on the progression and metastasis of gastric cancer [29–31]. Despite significant advancements in research, there are still gaps in our understanding of the intricate molecular pathways and regulatory networks involved in gastric carcinogenesis. The key finding of our study is that the lncRNA WFDC21P was upregulated in gastric cancer tissues and cell lines. WFDC21P downregulation using siRNAs inhibited gastric cancer tumor growth and metastasis. Further systematic researches revealed that WFDC21P interacted with SEC63 to regulate the calcium homeostasis signaling pathway to facilitate gastric cancer proliferation and metastasis. This finding solidified the pivotal role of lncRNAs in in the processes of tumorigenesis and cancer progression.

LncRANs were once regarded as byproducts or transcription noise during gene expression [32]. In recent years, lncRNAs have been reported to play an important role in human physiological and pathophysiological processes, especially in breast [33], lung [34], liver cancer [35]. In this study, online analysis tool GEO2R was used to screen the differential expressed lncRNAs in GEO database. Gastric cancer microarray datasets GSE53137, GSE58828, and GSE109476 were used in this study. After bioinformatic screening, lncRNA WFDC21P attracted our attention. It was reported that WFDC21P participated in serval types of cancer. As shown in previous studies, WFDC21P attenuates hepatocarcinogenesis via modulating glycolysis [36]. On the opposite side, studies showed that WFDC21P promoted lung [37], breast [38], and colorectal tumor [39] malignant behaviors. In this study, WFDC21P was upregulated in both gastric cancer tissues and cell lines. Through loss-of-function studies, the results of CCK-8, wound healing, and Transwell assays showed that WFDC21P downregulation inhibited tumor proliferation and metastasis significantly.

Accumulating evidence suggests that non-coding RNAs (ncRNAs) interacted with miRNAs or proteins to act as molecular sponge [40, 41]. In previous studies, WFDC21P was reported to bind with miR-4293 [37] and miR-628 [38] to regulate lung and breast cancer progression respectively. On the other aspect, studies showed that WFDC21P was activated by transcription factor Nur77 [36]. To further investigate the molecular mechanism of WFDC21P, catRAPID database was used to screen potential proteins that interacted with WFDC21P. Interestingly, SEC63 was found to interact with WFDC21P at the highest Interaction Propensity and Z score. After literature review, The SEC63 protein, also known as Sec63p in yeast, was reported mainly located at ER [42]. SCE61, SEC62 and SEC63 work together to transport immature proteins across ER membrane [43]. SEC63 have the capability to interact with chaperones to regulate proteins translocation [44]. Many studies have reported that SEC63 participated in human physiological process as well as tumorigenesis and progression [45]. However, whether SEC63 regulate gastric tumor growth and metastasis remains unknown. In this study, online bioinformatic tool was used to predict that SEC63 interacted with lncRNA WFDC21P. And this result was verified by RIP and RNA pull down assay. Further studies identified that WFDC21P was the upstream regulator of SEC63. Take the molecular function of SEC63 and WFDC21P together into consideration, WFDC21P was found to interacted with SEC63 to participate in calcium homeostasis regulation. Tumor phenotype assay revealed that SEC63 overexpression rescued the tumor suppressive effect caused by WFDC21P silencing. Above results showed that WFDC21P participated in regulating proteins translocation and maintaining calcium homeostasis in ER. Notably, there still have limitations in this study: the upstream regulator of WFDC21P remained unknown. This study has revealed that WFDC21P regulated calcium homeostasis, however, whether WFDC21P interacted with SEC63 to affect protein translocation in ER membrane remain to be further investigated. Further investigation about WFDC21P in endoplasmic reticulum stress regulation is required.

## Conclusions

**Fig. 5:**
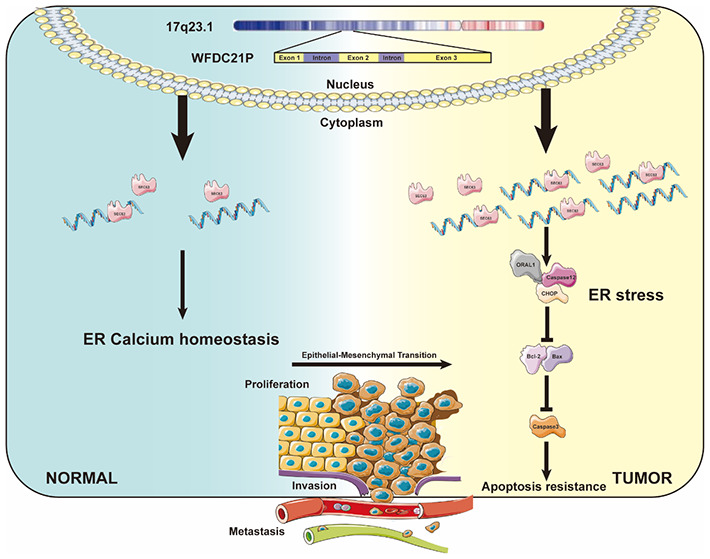
Proposed model of the molecular mechanism of WFDC21P in gastric cancer

To the best of our knowledge, this study was the first to confirmed that lncRNA WFDC21P interacted with SEC63 to promote gastric cancer malignant behaviors by regulating calcium homeostasis signaling pathway. Our results suggested that the WFDC21P and SEC63 may have prognostic and therapeutic applications in patients with gastric cancer (Fig. 5).

### Electronic supplementary material

Below is the link to the electronic supplementary material.

Supplementary Material 1

## Data Availability

All data generated or analyzed during this study are included in this published article.
