# Supplementary material for: LncRNA WFDC21P interacts with SEC63 to promote gastric cancer malignant behaviors by regulating calcium homeostasis signaling pathway
Source: Cancer Cell Int. 2024 Mar 25;24:111. doi: 10.1186/s12935-024-03297-2 (PMC10962109; doi:10.1186/s12935-024-03297-2)
Supplement: Supplementary file 1 — Supplementary Material 1 [file 12935_2024_3297_MOESM1_ESM.pdf]

Table S1. Sequences of qRT-PCR primers or siRNA used in this study.

Gene	Forward primer (5'-3')<br>Or Sense sequence	Reverse primer (5'-3')<br>Or Antisense sequence
WFDC21P	GGAGACCACTGTGTCAGCAA	ACTCCAGGAAGGGATGACGA
SEC63	CTTCGTGGGGCTCATCGTG	TCGAATTTGCTCGGCATTCT
siSEC63-#1	CCAGCCAAAUAUUAUUCCUACAG UA	UACUGUAGGAAUAAUAUUUGGCU GG
siSEC63-#2	UGGAAUUGCCCUGCCAGCUUGGA UA	UAUCCAAGCUGGCAGGGCAAUUC CA
siSEC63-NC	CCAAACAUAAUUCUUAUCACGCG UA	UACGCGUGAUAAGAAUUAUGUUU GG
siWFDC21P-#1	GCUUUCUGAAACGUUGUUCAGUC UA	UAGACUGAACAACGUUUCAGAAA GC
siWFDC21P-#2	UGAAACGUUGUUCAGUCUAGAUG AA	UUCAUCUAGACUGAACAACGUUU CA
siWFDC21P-NC	GCUCUGAAACGUUGUUCAGUUUC UA	UAGAAACUGAACAACGUUUCAGA GC
GAPDH	CACCCACTCCTCCACCTTTG	CCACCACCCTGTTGCTGTAG

Table S2. Antibodies used in Western blotting and RIP

Antibody	Company	Cat No.	Dilution
SEC63	Proteintech	13978-1-AP	1:2000for WB<br>2 ug for RIP
β-actin	Proteintech	20536-1-AP	1:2000
E-cadherin	Proteintech	20874-1-AP	1:5000
N-cadherin	Proteintech	22018-1-AP	1:5000
SNAIL1	Proteintech	13099-1-AP	1:500
HRP-labeled Goat Anti-Rabbit IgG(H+L)	Beyotime	A0208	1:1000
HRP-labeled Goat Anti-mouse IgG(H+L)	Beyotime	A0216	1:1000

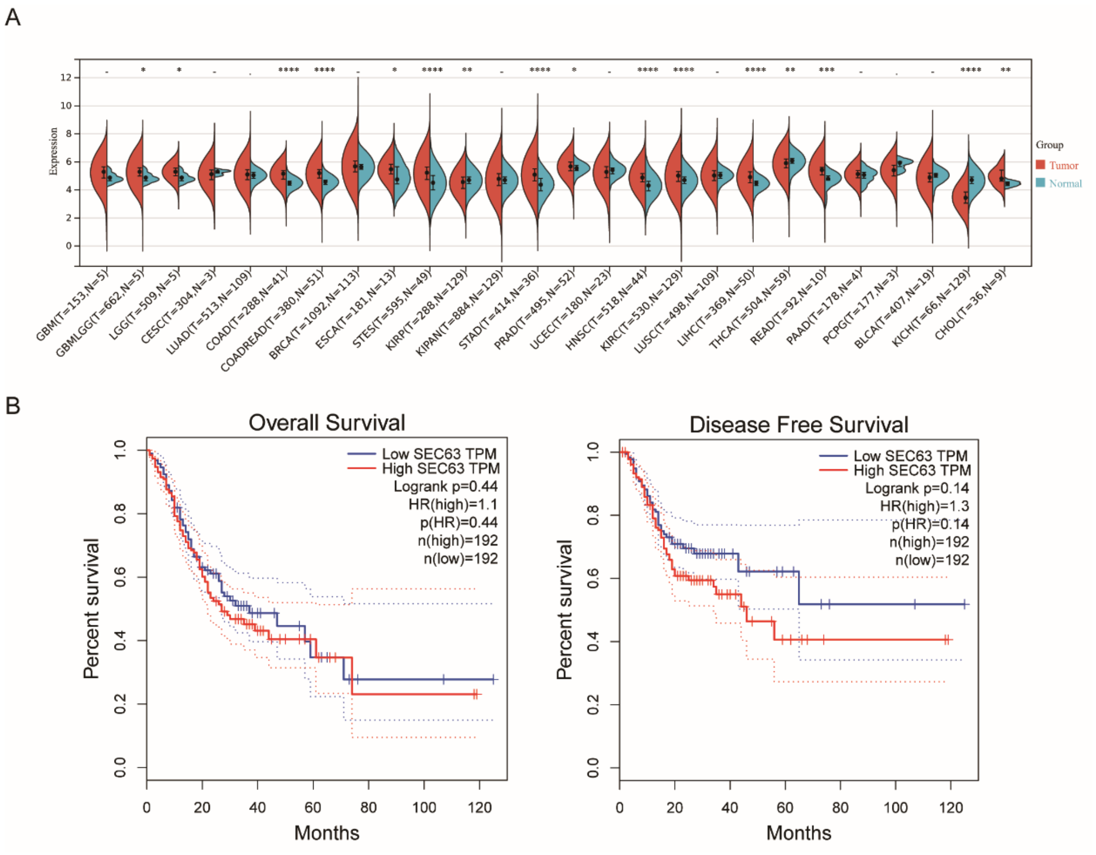

Fig. S1. The expression of SEC63 in gastric cancer.

A. Pan-cancer analysis was performed to investigate the expression of SEC63 in different types of cancer. **B.** K-M plot showed the relationship between SEC63 expression and patient prognosis.
